# Effect of Obesity on Perioperative Outcomes Following Lung Cancer Surgery: Protocol for a Meta-Analysis and Systematic Review

**DOI:** 10.2196/76315

**Published:** 2025-11-12

**Authors:** Qiuxiang Wang, Zhishu Li, Xihuan Wang, Bin Li, Chunfeng Wang, Yongguo Xiang

**Affiliations:** 1 Department of Traditional Chinese Medicine Guangyuan Central Hospital Guangyuan, Sichuan Province China; 2 Department of Respiratory Medicine Guangyuan Central Hospital Guangyuan China

**Keywords:** obesity, lung cancer, perioperative outcomes, surgery, meta-analysis, protocol

## Abstract

**Background:**

Surgical resection is the primary curative treatment for early-stage lung cancer—the leading global cause of cancer mortality, responsible for nearly 1 in 5 cancer deaths in 2022. Obesity is a global health concern that may influence surgical outcomes; yet, its impact on perioperative outcomes following lung cancer surgery remains controversial.

**Objective:**

This protocol outlines a meta-analysis and systematic review to evaluate the association between obesity and perioperative outcomes in patients who underwent a lung cancer resection.

**Methods:**

Observational studies related to patients with lung cancer who underwent surgical resection were searched in 5 English and 3 Chinese literature databases: PubMed, Embase, Cochrane Library, Web of Science, MEDLINE, Chinese National Knowledge Infrastructure, Wanfang, and the Chinese Biomedical Database. The search period for these 8 electronic databases was from inception to 2025. The PROSPERO database and the International Platform of Registered Systematic Review and Meta-Analysis Protocols (INPLASY) database were also searched. Qualified studies were screened and selected by 2 authors independently. The literature obtained were imported into NoteExpress to screen the titles and abstracts. After reading the full text of the remaining studies, the final number of studies were determined. Two reviewers independently extracted data from the included studies by using a predesigned data extraction tool. The Newcastle-Ottawa Scale was used to evaluate the quality of the research. The primary outcome of this study was to evaluate the postoperative mortality in people living with obesity undergoing lung cancer surgical procedures. The secondary outcomes were the postoperative complications, average length of stay, blood loss during the operation, and operation time in people living with obesity undergoing lung cancer surgical procedures. For dichotomous data, we plan to present results as risk ratios with 95% CIs. For continuous data, we will use mean difference with 95% CIs. The Review Manager software (version 5.4) will be used for the meta-analysis and statistical analysis. Sensitivity analysis and Egger test will be performed with Stata software (version 16.0).

**Results:**

The results are not yet accessible because this is a protocol for a systematic review and meta-analysis. The protocol is registered in PROSPERO under the registration number CRD42025648330. By August 26, 2025, we completed the literature search of the 8 databases and completed the selection and extraction of data.

**Conclusions:**

This study will synthesize existing evidence to clarify whether obesity is a risk factor for adverse outcomes or if it confers a protective effect, as suggested by the obesity paradox. These findings will guide clinical decision-making and improve perioperative care for obese people with lung cancer.

**Trial Registration:**

PROSPERO CRD42025648330; https://www.crd.york.ac.uk/PROSPERO/view/CRD42025648330

**International Registered Report Identifier (IRRID):**

PRR1-10.2196/76315

## Introduction

Lung cancer remains the leading cause of cancer-related mortality worldwide, with surgical resection serving as the primary curative treatment for early-stage non–small cell lung cancer [1,2]. Despite advancements in surgical techniques, pulmonary resection is still associated with significant perioperative risks, including respiratory complications, cardiovascular events, and mortality [3]. Consequently, identifying modifiable patient factors that influence the surgical risk is a critical focus of preoperative optimization. Although obesity is a well-established risk factor for numerous health conditions, its impact on lung cancer surgery outcomes presents a complex and often contradictory picture. The conventional obesity paradox, where a higher BMI is sometimes associated with improved survival in chronic diseases, conflicts with the known physiological challenges obesity imposes on respiratory and cardiac function [4,5]. This creates a significant dilemma for surgeons assessing preoperative risk. Therefore, elucidating the true relationship between obesity and postoperative complications is essential for refining risk stratification models and for improving surgical outcomes for patients with non–small cell lung cancer. Additionally, the presence of obesity complicates patient outcomes, often leading to poorer prognoses, increased treatment-related complications, and a higher likelihood of comorbid conditions [6]. Despite its association with comorbidities, the impact of obesity on perioperative outcomes in lung cancer surgery remains controversial. Some studies suggest that obesity may increase the risk of postoperative complications such as respiratory complications due to physiological stress and technical challenges during the surgery [7]. Conversely, other studies propose an obesity paradox, where obese people with BMI ≥30 exhibit better survival and fewer complications compared with normal weight and underweight patients following surgical resection of lung cancer [8]. This discrepancy highlights the need for a comprehensive synthesis of existing evidence.

The World Health Organization (WHO) defines overweight individuals as having BMI >25 kg/m^2^ and obese individuals as having BMI >30 kg/m^2^ [9]. Contemporary diagnostic frameworks such as those proposed by the European Association for the Study of Obesity [10] and The Lancet [11] advance the clinical conceptualization of obesity by emphasizing health impairments (eg, clinical obesity), and the Edmonton Obesity Staging System offers a powerful tool for mortality risk prediction [12]. The WHO BMI classification was selected as the principal typology for this study. This decision is grounded in the research's scope and objectives. The WHO criteria, with its clear BMI thresholds of >25 kg/m² and >30 kg/m², provides an unambiguous and universally applicable metric for categorizing participants. This is essential for ensuring consistency in a large-scale analysis and for enabling direct comparisons with the majority of existing oncological and public health studies that also rely on this standard. Therefore, the WHO definition serves as the most appropriate and practical foundation for this epidemiological investigation.

Obesity is recognized as one of the most important global public health problems, with an increase in its prevalence in almost all countries, rising as high as 2-fold in 70 countries since 1980 [13]. The global obesity epidemic, attributed to sedentary lifestyles, unhealthy diets, genetics, and environmental factors, has led to over 1.9 billion adults being classified as overweight and 650 million living with obesity [14]. Over a third of the global population is currently classed as overweight or obese, with projections suggesting that by 2030, 38% of the adults will be overweight and 20% will develop obesity [6]. This will pose specific challenges for surgeons who must address the physical, technical, and physiological problems associated with thoracic operations in patients living with obesity.

A meta-analysis found a significant positive association between excess body weight and the risk of lung cancer, particularly among nonsmokers and women [6]. One research found that obesity does not increase the perioperative complications in older patients undergoing thoracoscopic anatomic lung cancer surgery [15]. Ferguson et al [16] showed that being overweight or obese did not increase the risk of postoperative complications in any category after a major lung resection. Guerrera et al [17] evaluated the impact of morbid obesity on perioperative clinical outcomes after thoracoscopic lobectomy and found that people living with obesity did not show increased conversion rates, blood loss, and surgical time. Obesity is associated with improved overall survival in patients with non–small cell lung cancer after curative resection when skeletal muscle mass and radiodensity are preserved [18].

This protocol describes a planned meta-analysis to systematically evaluate the effect of obesity on the perioperative outcomes following lung cancer surgery. The primary outcome is postoperative 30-day mortality; the secondary outcomes are postoperative complications, average length of stay, blood loss during operation, and operation time. This study aims to provide evidence-based insights to guide preoperative risk assessment and optimize patient care. The study “Causes for Retraction in the Biomedical Literature: A Systematic Review of Studies of Retraction Notices” [19] is helpful for us to understand the common methodological defects in medical research, which could affect the quality of our meta-analysis research. This knowledge will strengthen our evaluation of the Newcastle-Ottawa Scale (NOS) [19].

## Methods

### Study Registration

This study is conducted in accordance with the PRISMA-P (Preferred Reporting Items for Systematic Reviews and Meta-Analyses Protocols) statement guidelines [20] and has been registered with PROSPERO under the registration number CRD42025648330. Any amendments to this protocol will be documented with a date and a brief rationale. All amendments will be updated in the PROSPERO registry to maintain transparency. The final review manuscript will explicitly report and justify any deviations from the original registered protocol.

### Criteria for the Studies

#### Types of Studies

Observational studies, including cross-sectional, case-control, or cohort studies, are the focus of this meta-analysis. Case reports, case series, review articles, letters, editorials, and commentaries are excluded. The language of the studies has a restriction of English or Chinese.

#### Types of Participants

People aged 18 years or older living with obesity diagnosed with lung cancer who underwent surgical resection are included in this study. Obesity is classified by WHO BMI of >30 kg/m^2^ in Western populations and >25 kg/m^2^ in Asian populations. We also use the Edmonton Obesity Staging System to assess obesity severity based on comorbidities and functional status and not just BMI. We present results for obese and overweight groups individually to allow for comparison and identify potential differential effects.

#### Types of Interventions

The intervention measures are video-assisted thoracoscopic surgery or open lobectomy of obese patients diagnosed with lung cancer.

#### Types of Comparisons

Nonobese patients diagnosed with lung cancer who underwent video-assisted thoracoscopic surgery or open lobectomy were considered as the control group.

#### Types of Outcome Measures

##### Primary Outcomes

The primary outcome considered is the postoperative 30-day mortality in patients living with obesity undergoing lung cancer surgical procedures.

##### Secondary Outcomes

The second outcomes are postoperative complications, pulmonary complications, cardiovascular complications, average length of stay, the blood loss during the operation, and the operation time in people living with obesity undergoing lung cancer surgical procedures.

### Exclusion Criteria

Studies that report the following are excluded.

1. Those that did not include a nonobese reference group.
2. Inconsistencies between the data reported in the manuscript and data represented in tables such as numerical mismatches between text and tables and conflicting results within the manuscript.
3. Studies with overlapping cohorts.

### Information Sources

A systematic search was performed in the following 5 English and 3 Chinese literature databases with a restriction of time from inception to 2025 to filter the eligible studies: PubMed, Embase, Cochrane Library, Web of Science, MEDLINE, Chinese National Knowledge Infrastructure, Wanfang, and the Chinese Biomedical Database.

### Search Strategy

The following search algorithm and Medical Subject Headings (MeSH) terms were used: [(lung cancer OR lung neoplasm) AND (video-assisted thoracoscopic surgery OR pulmonary surgical procedures) AND (body mass index OR obesity OR obese)]. We considered the specific search strategy in PubMed as a typical example, and the specific steps of the retrieval are shown in Textbox 1.

**Search strategy in PubMed database.**

**Search items**

#1: ((((((((Lung Neoplasms[MeSH Terms]) OR (Neoplasms, Pulmonary[Title/Abstract])) OR (Neoplasm, Pulmonary[Title/Abstract])) OR (Pulmonary Neoplasm[Title/Abstract])) OR (Lung Neoplasm[Title/Abstract])) OR (Lung Cancers[Title/Abstract])) OR (Pulmonary Cancer[Title/Abstract]))

#2: ((((Thoracic Surgery, Video-Assisted[MeSH Terms]) OR (Video-Assisted Thoracic Surgeries[Title/Abstract])) OR (VATSs[Title/Abstract])) OR (Video Assisted Thoracic Surgery[Title/Abstract]))

#3: (pulmonary surgical procedures[MeSH Terms]) OR (Pulmonary Surgical Procedure[Title/Abstract])

#4: #2 or #3

#5: ((Body Mass Index[MeSH Terms]) OR (Quetelet Index[MeSH Terms])) OR (Quetelet Index[MeSH Terms])

#6: Obesity[MeSH Terms]

#7: #5 or #6

#8: #1 and #4 and #7

### Data Collection and Analysis

#### Study Selection

Qualified studies were screened and selected by 2 independent authors. The literature was imported into NoteExpress to screen the titles and abstracts. After that, we obtained full-text articles of the relevant studies. After reading the full text of the remaining studies, the final included studies were determined. Any disagreements were arbitrated by a third reviewer.

#### Data Extraction

Data were extracted independently by 2 reviewers into a prepiloted, standardized electronic data collection form created in Microsoft Excel. Any disagreements were resolved by negotiation and discussion. Further controversy was arbitrated by a third reviewer. The following information was extracted. The detailed characteristics of the included studies, the first author’s name, published year, country, sample size, age, surgical approach, and tumor staging were extracted. We included studies with the primary outcome of postoperative 30-day mortality and secondary outcomes of postoperative complications, pulmonary complications, cardiovascular complications, total hospital stay, and operation time. Potential confounders (smoking status, comorbidities, cancer staging, surgical duration) were also collected. The GRADE (Grading of Recommendations Assessment, Development and Evaluation) method was used to evaluate the evidence quality of each main outcome index, and its certainty level was clearly reported to ensure the clinical credibility and interpretability of the systematic evaluation conclusion. Results were disseminated via open-access publications, conference presentations, PROSPERO updates, and public summaries for nonacademic stakeholders, with data upon request.

#### Quality Assessment

NOS was used to evaluate the quality of the research [21]. The NOS provides a checklist of items for evaluating the quality of reporting and the risk of bias of the included studies based on 3 broad evaluation categories: selection, comparability, and exposure/outcomes [22]. The scale has 3 parameters and 8 items with a total score of 9; scores ≤3 are usually considered low quality, scores of 4 or 5 are considered medium quality, and scores ≥6 are usually considered high quality [23]. Two reviewers will independently evaluate the quality of the included studies, and the third team member will verify them. Any differences will be settled through negotiation. To strengthen rigor, we integrated NOS scores into the analysis. Studies scoring ≤3 were excluded. Higher-quality studies (≥6) received greater weighting in the meta-analysis. The GRADE approach was adopted to systematically assess the certainty of evidence for each key outcome. Risk of bias, inconsistency, indirectness, imprecision, and publication bias were evaluated, after which the overall certainty was classified as high, moderate, low, or very low.

#### Measures of Treatment Effect

For dichotomous data, we plan to present results as risk ratios with 95% CIs. For continuous data, we will use mean difference with 95% CIs. *P* values less than .05 were considered as statistically significant.

#### Data Analysis and Heterogeneity Processing

The Review Manager software (version 5.4) will be used for meta-analysis and statistical analysis. Sensitivity analysis and Egger test will be performed with Stata software (version 16.0). Because patients who were operated at different centers may have varying risk profiles and varying selection criteria for each surgical technique, the random effects model will be chosen to take this heterogeneity into account thus has a more conservative value [24,25]. *P*<.05 will be considered statistically significant. Significant heterogeneity will be characterized by *I*^2^>50% and/or *P*<.01. Besides, the levels of heterogeneity will be assessed by *I*^2^ as follows: 0%-25% indicates homogeneity, 25%-50% indicates low heterogeneity, 50%-75% indicates moderate heterogeneity, and >75% indicates high heterogeneity [26]. To address heterogeneity from differential BMI classifications, we will stratify the meta-analysis by geographic region (Asian vs Western populations) by using their respective standard BMI thresholds. Pooled estimates will be calculated separately for each subgroup.

#### Subgroup Analysis

Subgroup analyses will be conducted based on the degree of obesity, type of surgical procedure, and geographic region. To mitigate the risks of post hoc subgrouping and interpretive bias, we will prespecify all the subgroups in the analysis plan. We will clearly define obesity severity by standard BMI thresholds, region by geographic or administrative boundaries, and surgical method by specific procedural codes.

#### Sensitivity Analysis

The stability of the meta-analysis results will be tested through a one-by-one elimination method in the sensitivity analysis. Studies were stratified according to both WHO and region-specific BMI criteria. Sensitivity analyses will be conducted to assess the impact of different BMI thresholds by region on the pooled estimates, thereby ensuring comparability and minimizing potential bias.

#### Assessment of Reporting Bias

When we select >10 studies consistent with conditions, a funnel plot will be used to detect the publication bias, while the Egger test of bias will be used as a supplement.

#### Dealing With Missing Data

Should clarification or additional data be necessary from the included studies, the corresponding authors of these studies will be contacted via telephone or email. Duplicate reporting across studies (registry-based publications) will not be screened.

### Ethical Considerations

This systematic review and meta-analysis will be based on published data. As researchers did not access any information that could lead to the identification of an individual patient, no concerning ethical issue was raised in this research. Therefore, obtaining ethical approval and consent of participants was waived. Research results may be published in a peer-reviewed journal or disseminated in relevant conferences.

## Results

**Figure 1:**
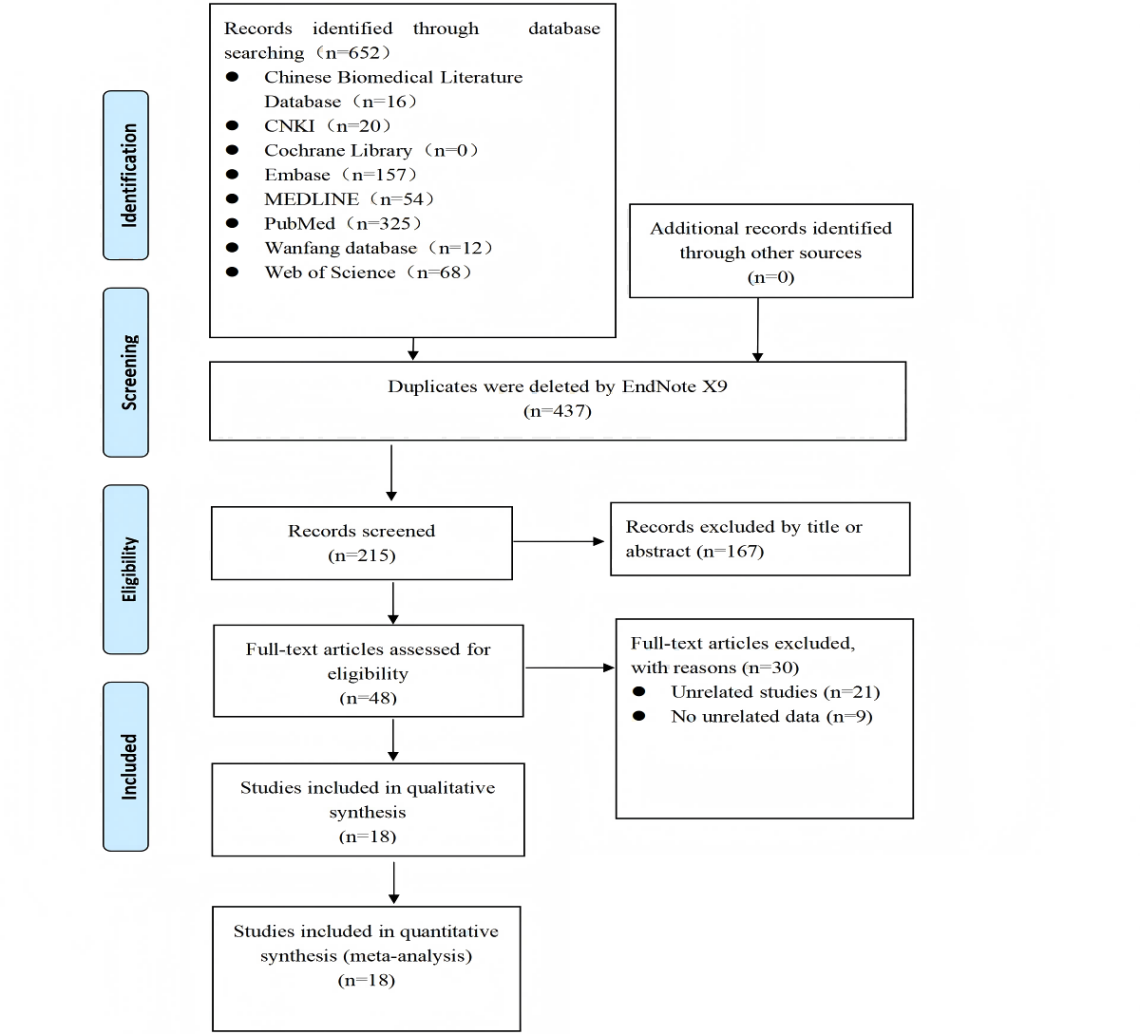
PRISMA (Preferred Reporting Items for Systematic Reviews and Meta-Analyses) flow diagram. CNKI: Chinese National Knowledge Infrastructure.

Results are not yet available because this is a protocol for a systematic review and meta-analysis. This protocol was registered on PROSPERO on February 5, 2025. By August 26, 2025, we completed the literature search of 8 databases, study selection, and data extraction from the selected studies. The specific analysis has not been completed yet. The search and selection process is illustrated in the flow diagram in Figure 1. The results are expected to be published in March 2026.

## Discussion

This meta-analysis aims to synthesize existing evidence to elucidate the relationship between obesity and perioperative outcomes following lung cancer surgery, providing a comprehensive understanding of the risks and implications for clinical practice. These findings will contribute to a better understanding of the obesity paradox in surgical oncology and may inform preoperative risk stratification and patient counseling. A more insightful analysis would critically examine the methodological heterogeneity across these studies, such as differences in patient cohorts (eg, types of surgery, cancer stages) and, most importantly, the variable adjustments for potent confounders such as smoking, cachexia, or cardiorespiratory fitness. Discrepancies in these elements are likely a primary source of the apparent paradox and warrant explicit elaboration.

The methodological rigor of this meta-analysis, including a comprehensive search strategy, strict inclusion criteria, and robust statistical analysis, will enhance the reliability of the findings. By stratifying results based on BMI categories and adjusting for confounding variables such as age, smoking status, and comorbidities, this study will offer nuanced insights into the impact of obesity on perioperative outcomes. Furthermore, subgroup analyses based on the surgical approach (eg, open vs minimally invasive) and cancer stage will help identify specific populations at higher risk.

The findings of this meta-analysis have important implications for preoperative risk assessment and patient counseling. If the obesity paradox is confirmed, it may prompt a reevaluation of current risk stratification models and highlight the need for further research into the underlying mechanisms. Osteoarthritis research frequently encounters the obesity paradox, where patients with higher BMI sometimes show unexpected outcomes [27]. This could provide insights into how other researchers have approached similar contradictory findings in their analyses.

In conclusion, this meta-analysis will provide valuable evidence on the effect of obesity on the perioperative outcomes following a lung cancer surgery. By addressing the inconsistencies in the existing literature and exploring potential moderating factors, this study will contribute to the optimization of surgical care for obese people with lung cancer.

## Appendix

Multimedia Appendix 1 PRISMA-P checklist.

## Abbreviations

GRADE: Grading of Recommendations Assessment, Development and Evaluation
MeSH: Medical Subject Headings
NOS: Newcastle-Ottawa Scale
PRISMA-P: Preferred Reporting Items for Systematic Reviews and Meta-Analyses Protocols
WHO: World Health Organization

## Data Availability

All data generated or analyzed during this study are included in this published article and are available from the corresponding author on reasonable request.
